# Population genetics at three spatial scales of a rare sponge living in fragmented habitats

**DOI:** 10.1186/1471-2148-10-13

**Published:** 2010-01-14

**Authors:** Andrea Blanquer, Maria J Uriz

**Affiliations:** 1 Departament d'Ecologia Marina, Centre d'Estudis Avançats de Blanes, CSIC. Accés Cala St Francesc, 14. 17300 Blanes, Girona, Spain

## Abstract

**Background:**

Rare species have seldom been studied in marine habitats, mainly because it is difficult to formally assess the status of rare species, especially in patchy benthic organisms, for which samplings are often assumed to be incomplete and, thus, inappropriate for establishing the real abundance of the species. However, many marine benthic invertebrates can be considered rare, due to the fragmentation and rarity of suitable habitats. Consequently, studies on the genetic connectivity of rare species in fragmented habitats are basic for assessing their risk of extinction, especially in the context of increased habitat fragmentation by human activities. Sponges are suitable models for studying the intra- and inter-population genetic variation of rare invertebrates, as they produce lecitotrophic larvae and are often found in fragmented habitats.

**Results:**

We investigated the genetic structure of a Mediterranean sponge, *Scopalina lophyropoda *(Schmidt), using the allelic size variation of seven specific microsatellite loci. The species can be classified as "rare" because of its strict habitat requirements, the low number of individuals per population, and the relatively small size of its distribution range. It also presents a strong patchy distribution, philopatric larval dispersal, and both sexual and asexual reproduction. Classical genetic-variance-based methods (AMOVA) and differentiation statistics revealed that the genetic diversity of *S. lophyropoda *was structured at the three spatial scales studied: within populations, between populations of a geographic region, and between isolated geographic regions, although some stochastic gene flow might occur among populations within a region. The genetic structure followed an isolation-by-distance pattern according to the Mantel test. However, despite philopatric larval dispersal and fission events in the species, no single population showed inbreeding, and the contribution of clonality to the population makeup was minor (only ca. 4%).

**Conclusions:**

The structure of the *S. lophyropoda *populations at all spatial scales examined confirms the philopatric larval dispersal that has been reported. Asexual reproduction does not seem to play a relevant role in the populations. The heterozygote excess and the lack of inbreeding could be interpreted as a hitherto unknown outcrossing strategy of the species. The envisaged causes for this strategy are sperm dispersal, a strong selection against the mating of genetically related individuals to avoid inbreeding depression or high longevity of genets combined with stochastic recruitment events by larvae from other populations. It should be investigated whether this strategy could also explain the genetic diversity of many other patchy marine invertebrates whose populations remain healthy over time, despite their apparent rarity.

## Background

Many marine invertebrates are sessile as adults, have limited dispersal abilities, and their populations are in general well-structured (e.g., [1-8]), despite the fact that marine environments have long been considered to be more open than terrestrial environments [9]. Genetic exchange (i.e., gene flow) among populations of sessile invertebrates is restricted to the dispersal of gametes, and asexual and/or sexual propagula (often lecitotrophic, short-living, small larvae).

The particular rocky habitats of most marine sessile invertebrates (i.e., sponges, cnidarians, brooding bryozoans and ascidians) are often fragmented in space. Habitat fragmentation has been reported to account for population isolation in invertebrates with poor dispersal capabilities through the hampering of genetic exchange among populations [10]. Moreover, at long temporal scales, fragmented habitats can promote species rarity [11]. Consequently, fragmented habitats may either put the survival of the species at risk or trigger genetic, biological and/or ecological strategies to overcome the problems that arise from isolation. At evolutionary time-scales, fragmentation of habitats can also favor speciation (e.g., [12,13]).

Rare species are considered to be strongly vulnerable in fragmented habitats [14], especially when gene flow among patches is restricted or absent. Most definitions of the rarity of species include ideas about habitat requirements, the number of individuals, and the size of distribution ranges, which can vary over temporal and spatial scales [15]. The status of "rare" species is especially difficult to assess appropriately for marine invertebrates, as there is a lack of quantitative data in these respects [11,14]. Accordingly, many cryptic, patchily-distributed marine invertebrates are not considered rare because of assumed inappropriate sampling and the incorrect assumption that marine distant populations are always connected through the exchange of pelagically dispersing propagula [16,17]. Since habitat fragmentation can be aggravated by human perturbations, studies on the genetic connectivity of rare species in fragmented habitats are particularly necessary for the assessment of their extinction risk, and this should be taken into account when developing management rules or for conservation issues.

Sponges are key, widespread elements in benthic ecosystems (e.g., [18,19]). Thus, they represent suitable models for studying inter-population connectivity, the colonization of new habitats, and intra- and inter-population genetic variation in sessile marine invertebrates. However, despite their ecological importance, very little is known about the genetic issues of sponge populations such as gene flow, genetic variation, and effective dispersal. Therefore, genetic studies on sponges are still largely lacking.

*Scopalina lophyropoda *(Schmidt, 1862) has only been recorded from a few Western Mediterranean localities [20-24]. Recent reports from Southern and Eastern Mediterranean localities [25,26] need genetic confirmation, because cryptic species have been recently reported [13,27]. The species only dwells on the lower zones of north-facing rocky walls and blocks (i.e., close to the bottom), in areas with a certain amount of suspended organic material (e.g., close to a river mouth) [13,28]. The species habitat is strongly fragmented along the species distribution area. After performing exhaustive samplings at distances shorter than 100 km, with sub-samplings at each sampling site, we can state that *S. lophyropoda *can be classed as a "rare species" according to the definition of Chapman [11], owing to its strict habitat requirements, the low number of individuals per population, and the relatively small size of its distribution range: the well-known Western Mediterranean sublittoral [13].

Several known reproduction traits of *S. lophyropoda *allowed us to predict some aspects of its population genetics. First, the larvae do not move far away from the release point, but remain close to their parents in a vertical posture, suggesting philopatry [22,28,29]. Second, asexual reproduction by fission [30] indicates that clonality may play some role in determining the genetic makeup of populations. Third, the release of asexual propagula containing several mature larvae [31] may represent a stochastic dispersal event. A previous study on the species at a small spatial scale (from centimeters to tens of meters) using molecular markers confirmed the restricted dispersal of philopatric larvae, but revealed that the extent of clonality was low [32].

The main objective of this study was to gain knowledge on the genetic connectivity among populations of a marine rare sponge species inhabiting fragmented habitats (*S. lophyropoda*) in order to better understand the maintenance and growth of its populations, which have been monitored for the last 20 years. The study was performed at three spatial-scales: within populations, between populations of a geographic region, and between isolated regions. We also assessed the relative extent of sexual and asexual reproduction in the populations.

The knowledge acquired on this sponge species may be of interest in studies dealing with those rare modular invertebrates with limited dispersal, and whose habitats are disrupted, which are frequent in benthic ecosystems [11].

## Results

### Microsatellite polymorphism across loci

All of the amplifications were visualized on agarose gels and produced positive bands. However, despite good amplifications, double checking, and repetitions, the sizes of the alleles could not be estimated properly from the electropherograms for five individuals (ca. 2%) for locus Scol_d, three individuals (ca. 1%) for Scol_n and one (ca. 0.5%) for both, Scol_p and Scol_c. These unsized alleles were not included in the analyses, but cannot be considered null alleles. All microsatellite loci were polymorphic (Table 1). A total of 46 alleles were detected over the seven loci, with a range from 4 (Scol_c, Scol_i and Scol_p) to 12 (Scol_l) alleles per locus. There were private alleles in all populations except FE (probably due to the low number of individuals in this population; Table 2). A third of all of the linkage disequilibrium tests revealed linkages between pairs of loci, but these linkages were not consistent across populations. In each population, all loci behaved as neutral according to the Ewens-Watterson test. All loci, but Scol_l and Scol_n, showed negative values for the inbreeding coefficient (F_IS_; Table 1).

**Table 1:** Summary of statistics for the *Scopalina lophyropoda *microsatellites


**Scol_c**	4	0.4932	0.4417	-0.173*
**Scol_d**	4	0.7834	0.808	-0.076
**Scol_i**	8	0.1486	0.1411	-0.071
**Scol_l**	12	0.6036	0.6578	0.013
**Scol_n**	8	0.4155	0.5698	0.112
**Scol_p**	4	0.1991	0.1862	-0.098*
**Scol_r**	6	0.3378	0.3187	-0.169*


A = the total number of alleles per locus; the observed (H_O_) and expected (H_E_) heterozygosities and the inbreeding coefficients (F_IS_; *P ≤ 0.05) for each locus.

### Genetic diversity at different spatial scales

All the "a priori" defined populations of *Scopalina lophyropoda*, were genetically diverse (Table 2), with mean gene diversities (H_E_) ranging from 0.322 (GA) to 0.469 (SA). Single locus exact tests for the Hardy-Weinberg equilibrium indicated significant deviations (P < 0.05) in 9 out of 42 comparisons. In the MU population there was heterozygote deficit, whereas in all other populations the departures were due to heterozygote excesses (Table 2). Multi-locus fixation indexes, F_IS_, ranged from 0.017 in MU (the only positive value) to -0.099 in SA, and both were in the Blanes region. Multi-locus F_IS _values for all populations, but that in MU, were negative, although only values for CG, SA and GA were significantly different from 0 (P < 0.05), revealing outcrossing.

**Table 2:** Summary of statistics for the *Scopalina lophyropoda *microsatellites for each region and population site

Region	Location	N	Na	Pa	H_O_	H_E_	F_IS_
**CG**		**32**	**5.1**	**5**	**0.509**	**0.467**	-0.088*
	**CG**	32	5.1	5	0.509	0.467	-0.088*
							
**BL**		**97**	**5**	**2**	**0.469**	**0.465**	**-0.023**
	**MU**	40	4.4	1	0.443	0.450	0.017
	**SA**	43	4.3	1	0.515	0.469	-0.099*
	**FE**	14	3	0	0.432	0.406	-0.069
		**93**					
**PC**			**4.1**	**3**	**0.343**	**0.344**	**-0.016**
	**BA**	40	3	1	0.375	0.368	-0.018
	**GA**	53	3.9	2	0.346	0.322	-0.075*

N = the sample size; Na = the mean number of alleles per locus; Pa = the number of private alleles; the mean observed (H_O_) and expected heterozygosities (H_E_), and the inbreeding coefficients (F_IS_; *P ≤ 0.05).

**Figure 1:**
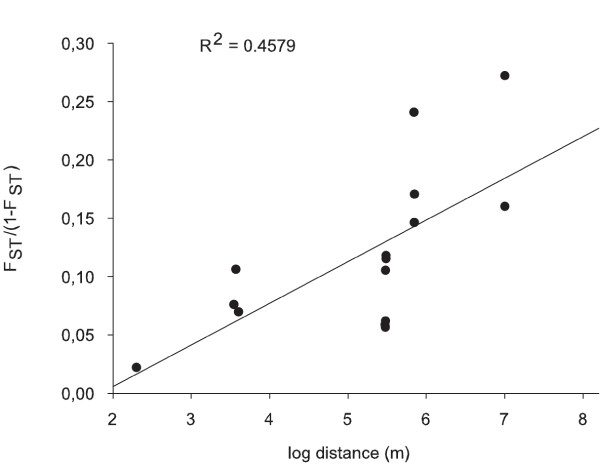
**Genetic isolation by distance of all the *Scopalina lophyropoda *populations as inferred using multi-locus estimates of F_ST_/(1-F_ST_) and the logarithm of the geographical distance (the Mantel test)**. The correlation coefficient R^2 ^= 0.45, p < 0.05.

The global estimates of F_ST _and R_ST_, over all populations, were significantly (P < 0.05) different from 0 (F_ST _= 0.122 and R_ST _= 0.076). Multi-locus pairwise F_ST _indicated significant differences among all the population pairs (Table 3A). Regression of F_ST_/(1-F_ST_) values against the logarithm of the geographic distances revealed significant isolation by distance according to the Mantel test (R^2 ^= 0.4579; P < 0.05, Figure 1).

**Table 3:** Pairwise multi-locus F_ST _values between *Scopalina lophyropoda *population pairs (A) and between geographical region pairs (B) (* P < 0.05).

	**CG**	**MU**	**SA**	**FE**	**BA**	**GA**		**CG**	**BL**	**PC**
	
**CG**	-						**CG**	-		
**Mu**	0.195*	-					**BL**	0.150*	-	
**SA**	0.127*	0.021*	-				**PC**	0.179*	0.058*	-
**FE**	0.144*	0.094*	0.066*	-						
**BA**	0.141*	0.094*	0.050*	0.051*	-		**B**			
**GA**	0.216*	0.105*	0.100*	0.053*	0.060*	-				
				
**A**										

As for the geographical regions (Table 2), Cabo de Gata Natural Park had the highest value of mean number of alleles (5.1) whereas Port Cros National Park had the lowest value (4.1). Mean expected heterozygosities were similar for Cabo de Gata and Blanes regions (0.467 *vs *0.465), but were slightly lower for Port Cros (0.344). Multi-locus pairwise F_ST _indicated significant differences among the geographical regions (Table 3B). The global F_ST _and R_ST _values among the regions (F_ST _= 0.104; R_ST _= 0.042) revealed the presence of significant genetic structure (P < 0.05).

Overall, the hierarchical AMOVA revealed significant genetic structuring of the analyzed samples at the three geographical levels (7.57% among regions, 4.66% among populations within regions; and 87.77% within populations; Table 4). The analyses revealed that most of the genetic variation was found within the populations.

**Table 4:** The results of the analysis of molecular variance (AMOVA) on *Scopalina lophyropoda *microsatellites.

Source of variation	d. f.	Sum of squares	Variance components	Percentage of variation	Fixation indices	Pvalue
Among regions	2	46.955	0.1207	7.57	0.076 F_CT_	P < 0.001
Among populations within regions	3	19.827	0.0744	4.66	0.050 F_SC_	P < 0.001
Within populations	438	613.166	139.992	87.77	0.123 F_ST_	P < 0.05
Total	433	679.948	159.499			

**Figure 2:**
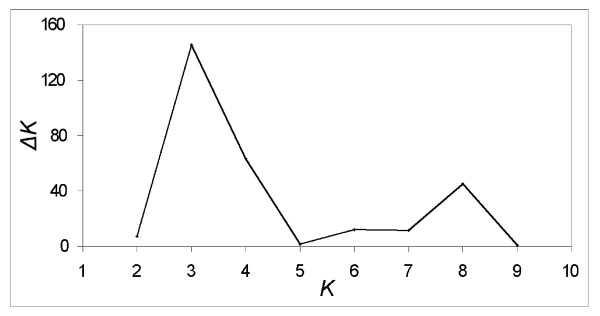
**Values of Δ*K *for each number of genetically homogeneous populations (*K*) considering all the *Scopalina lophyropoda *individuals**.

Bayesian clustering analyses detected the highest likelihood for the model with three genetically homogeneous groups of individuals (*K*), as shown by the highest peak in the Δ*K *for *K *= 3 (Figure 2), according to Evanno *et al*. (2005).

The Wilcoxon test detected recent bottlenecks in the CG, MU and GA populations (P two-tails values of 0.016, 0.0078 and 0.039, respectively), under the Stepwise Mutation Model (SMM). The tests performed, assuming the Two Phase model (TPM) and the Infinite Allele model (IAM), rendered non-significant results. With respect to the geographical regions, the Wilcoxon test again detected recent bottlenecks in each of the three regions (CG, BL, PC) using the SMM model (P two-tails values of 0.016, 0.023 and 0,039, respectively) but not using the TPM and IAM models.

### Contribution of clonal reproduction to the population structure

The number of multi locus genotypes (MLGs) occurring more than once was variable in the populations studied (Table 5). Individuals sharing MLGs were found in all populations except FE and SA. In Port Cros National Park, one MLG was shared by individuals from different populations: two from the BA population and one from the GA population (Table 5).

**Table 5:** Identical multi-locus genotypes (MLGs) that were detected in the *Scopalina lophyropoda *populations.

				Genotype							
					
Region	Pop.	MLG	N	Scol_c	Scol_d	Scol_i	Scol_l	Scol_n	Scol_p	Scol_r	MLGsim
CG	CG										
		1	2	146/**148**	313/**319**	**153/153**	**165**/169	**199**/201	**149**/**149**	118/**124**	*
		2	2	144/**148**	317/**319**	**153/153**	**165**/169	**199**/**199**	**149**/**149**	**124**/**124**	*
Bl											
	MU	1	2	146/**148**	317/317	**153/153**	**169**/**169**	**193**/**193**	**149**/**149**	**124**/126	ns
	SA	0									
	FE	0									
PC											
	BA	1	2	**148**/**148**	**313**/**313**	**153/153**	171/171	**193**/**193**	**149**/**149**	**124**/**124**	ns
		2	2	**148**/**148**	**313**/319	**153/153**	**169**/**169**	**193**/**193**	**149**/**149**	**124**/**124**	ns
		3	2	146/**148**	**313**/319	**153/153**	**169**/171	**193**/215	**149**/**149**	**124**/**124**	ns
		4	2	**148**/**148**	315/319	**153/153**	**169**/171	**193**/**193**	**149**/**149**	**124**/**124**	ns
		5	2	146/**148**	**313**/319	**153/153**	**169**/171	**193**/199	**149**/**149**	**124**/**124**	ns
		6	3	**148**/**148**	319/319	**153/153**	171/171	**193**/**193**	**149**/**149**	**124**/**124**	*
		7	4	146/**148**	**313**/319	**153/153**	**169**/**169**	**193**/199	**149**/**149**	**124**/**124**	*
	GA	1	2	**148**/**148**	**311**/313	**153/153**	**169**/**169**	**193**/**193**	**149**/**149**	**124**/**124**	ns
		2	2	**148**/**148**	**311**/313	**153/153**	**169**/171	199/215	**149**/**149**	**124**/**124**	ns
		3	2	**148**/**148**	315/315	**153/153**	171/171	**193**/**193**	**149**/**149**	120/**124**	*
		4	2	**148**/**148**	313/313	152/**153**	**169**/**169**	**193**/217	**149**/**149**	**124**/**124**	*
		5	3	**148**/**148**	**311**/313	**153/153**	171/171	**193**/**193**	**149**/**149**	**124**/**124**	ns
	BA+GA	1	2+1	146/**148**	**313**/319	**153/153**	**169**/171	**193**/**193**	**149**/**149**	**124**/**124**	ns

The genotypes are characterized using the allele sizes; N, the number of individuals sharing MLG; MLGsim, (* indicates significantly small probabilities of being the result of random mating; and ns, non-significance at P < 0.05). Numbers typed in bold indicate the allele with the highest frequency in the population.

The probabilities of finding identical MLGs by random mating were significantly small (P < 0.05) for the ramets from CG, for the groups of three and four ramets sharing MLGs in BA, and for two groups of two ramets presenting identical MLGs in GA (Table 5). A sexual origin for the two individuals in BA and the one in GA that shared MLG could not be rejected (P < 0.05). The probability of finding the latter MLG in three different genets was estimated by considering the allele frequencies in the whole Port Cros National Park region (the BA and GA populations).

## Discussion

Microsatellites have proven their use as powerful markers for population and clonality studies in the sponge *Scopalina lophyropoda*. The differentiation statistics used revealed that the genetic diversity was spatially structured in this rare species at the three spatial scales analyzed (as revealed by the F_ST _values and the AMOVA). Most diversity was found within the populations, but differentiation among populations and among geographic regions was also significant. The Bayesian clustering method (STRUCTURE), however, turned out to be more conservative and only detected three genetically independent groups, which correspond to the three geographical regions. However, when the polymorphism shown by the selected markers is limited, as it occurs in sponge microsatellites, or there is a strong isolation by distance pattern, the STRUCTURE algorithm is limited to resolve population clusters [33], and has been reported to provide a lower resolution than differentiation statistics [34-36]. Thus, considering all other results (Mantel test, AMOVA, F_ST _values, and the presence of private alleles), we can conclude that the sampled populations are genetically well structured, though some gene flow may take place within regions.

The positive regression between genetic differentiation and geographic distance between populations of this species revealed distance isolation and short-range effective dispersal. This pattern agrees with the philopatric behavior reported for *S. lophyropoda *larvae [28,29], which, in combination with the strict habitat requirement of the species [13], could contribute to its extremely fragmented distribution. Environmental stress is likely to be stronger in small fragmented populations than in large populations [37], and it has been reported to lead to rapid genetic divergence [38]. The later studies have suggested that the intensity and direction of the selection could differ among small populations [37] and lead to speciation. The presence of several cryptic *Scopalina *species in the distribution area of *S. lophyropoda *[27] could be interpreted in that way.

Gene flow can be maintained by dispersal of sexual and/or asexual propagules. In *S. lophyropoda*, the contribution of clonality to the populations was minor. Only 4% (9 out of 222) of the ramets analyzed can be assumed to be the result of asexual reproduction. A plausible explanation is that fissions are balanced with fusions between clone-mates [30], since clones remain attached to the rocky substrate and can contact each other when growing.

Since asexual dispersal appears to be unimportant in the species and the few clones identified use to be found at smaller distances than 0.5 m [32], gene flow between the populations depends on the dispersal of sexual products: either larvae, or sperm. Unfortunately, little is known about the dispersal of sponge sperm in general, and nothing is known about *S. lophyropoda *sperm in particular but the reported short-range dispersal [22,28,29,32] of *S. lophyropoda *larvae, fully agrees with the strong genetic structure in the species. The strong genetic structuring (AMOVA, F_ST _values and Mantel test results) indicates that there is a current absence of connectivity among the regions but STRUCTURE results point to some, although restricted, gene flow among the populations within a region, which may occur through sporadic stochastic dispersal events. A combined sexual-asexual dispersal mechanism consisting in asexual fragments of ripe individuals containing larvae has been speculated [31], which, if it occurs in the field, could enhance gene flow over longer distances than those suggested by the observed larval behavior in the field.

Bottleneck tests under IAM and TPM did not reveal any recent reduction in size of the populations, which is in agreement with the growth detected during the last 20 years in the several populations studied (authors' obs.). Only under the less probable mutation model for the microsatellites (SMM) [39-42], the bottleneck analysis indicated recent reductions in size in some populations. Although we do not have information on the mutation model of *S. lophyropoda *microsatellites, some studies in other taxa (e.g. humans, fish, insects) reported that microsatellites fit the mixed TPM or the IAM, but not the SMM [39-42]. Thus, field monitoring, the best models for microsatellite mutation, and the presence of private alleles in all populations (rare alleles are the first that disappear after a bottleneck), indicate that if any size reduction has occurred, it was not recently.

Most marine invertebrates with non-feeding and short dispersed larvae present genetically structured populations and inbreeding, whatever the Phylum they belong to [2,43-45]. As for sponges, only one study of population genetics using microsatellites is available up to now (*Crambe crambe*; [2]). Some population traits of *S. lophyropoda *are shared with those reported for *C. crambe*, whereas other aspects are contrasting. The populations are genetically structured in both species, despite their respective patchy *vs*. continuous distributions and the differences in larval behavior [28]. Restricted dispersal seems to be the main factor responsible for the isolation by distance that is found in the two sponges. However, the contribution of clonality to the population makeup was rather insignificant in *S. lophyropoda *(4% on average) while it reached 23% in *C*. *crambe *[2,28]. There are two possible explanations for the differences found between both species. First, in the study on *C. crambe*, several individuals did not amplify for each locus, which may contribute to a failure in differentiation of some individuals that were not real clones. On the other hand, fusion rates between ramets (also much more frequent in *S. lophyropoda *than in *C. crambe*) are more balanced in the former with fission rates and thus could account for the low extent of clonality in our "snapshot" sampling.

Moreover, all *C. crambe *populations showed inbreeding [2], as expected in long-lived, low-dispersing organisms due to temporary Wahlund effects [46,47]. Furthermore, individuals in small populations usually present inbreeding, which can increase their extinction probability by decreasing their potential for adaptation [39], especially when fragmented [14]. However, contrary to most predictions, we show here that *S. lophyropoda *has no inbreeding depression and exhibits heterozygote excess for most of the loci studied. In fact, our results contrast with theory and experimental evidences, which suggest that the effective size of small populations, and its response to selection, decreases with time [48]. The unexpected genetic results in the *S. lophyropoda *populations, however, support studies that predict that inbreeding should not be that common in marine invertebrates, despite their restricted dispersal, strong population structure, and consanguinity (e.g.,[49-51]). Our study provides indirect evidence on the existence of genetic strategies for maintaining the high genetic diversity even when the populations are isolated by distance and there is restricted gene flow among the populations. Selection against homozygote and/or for outcrossing, extreme genet longevity combined with stochastic recruitment events from other populations [51] are among the strategies that may allow *S. lophyropoda *and other rare, marine, benthic invertebrates to persist in a fragmented scenario.

## Conclusions

*Scopalina lophyropoda*, as other sessile marine invertebrates, *a priori *considered rare, lives without apparent constrains in fragmented populations with natural low abundances and restricted dispersal abilities. Mechanisms that are as yet poorly understood may be acting in those populations to maintain the necessary genetic diversity for the species to adapt to changing environments. This study has shown the results of these potential strategies, which would act in *S. lophyropoda *to avoid inbreeding, despite the species biological features. Efforts should be concentrated on the study of these mechanisms, which are responsible for the persistence of most marine sessile invertebrates with small, scattered populations and which have restricted dispersal, for their implications in the maintenance of marine biodiversity. Results on these mechanisms should be considered in the implementation of conservation directives, as rare species inhabiting fragmented habitats may require particular protection rules.

## Methods

### Sampling

**Figure 3:**
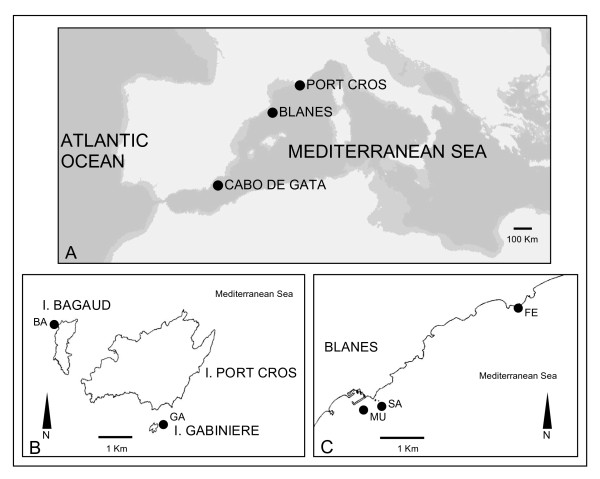
**Location of the *Scopalina lophyropoda *populations**. (A). Sampling sites at the Port Cros Park region (Ile de Bagaud, BA and Ilot de la Gabinière, GA; B), and at the Blanes region (Muntanyeta, MU, Santa Anna Niells, SA and Fenals, FE; C).

*Scopalina lophyropoda *populations were sampled by SCUBA diving from several localities in the Northwestern Mediterranean. The species identity was confirmed through amplification of a fragment of mtDNA *cytochrome c oxidase *gen, given the presence of several cryptic species [13,27]. Due to its rarity, after an exhaustive search, only 8 populations were found and sampled along the western Mediterranean. Samplings were performed every 100 km from the Adriatic to the South of the Iberian Peninsula and at the Atlantic Canary Islands. Populations from Banyuls-sur-Mer and Marseilles that yielded only 4 and 8 individuals, respectively, were genetically characterized, but excluded from the data analyses to avoid artifacts from the use of extremely unbalanced data. Consequently, the study included fragments from 222 individuals collected at 6 locations belonging to 3 distinct geographic regions (Figure 3A): Cabo de Gata Natural Park, Spain (CG), 36°51'N, 2°10'W; Port Cros National Park, France (PC, Figure 3B), 43°01'N, 6°23'E (Ile de Bagaud -BA- and Ilot de la Gabinière -GA-); and Blanes, Catalonia (BL, Figure 3C), 41°40'N, 2°47'E, (Muntanyeta -MU-, Santa Anna Niells -SA-, and Fenals -FE-); Table 2). Cabo de Gata Natural Park and Port Cros National Park are marine protected areas (MPA) and are therefore under special management conditions. Sponge fragments were preserved in absolute ethanol, which was changed three times, before storing at -20°C until DNA extraction.

### Microsatellite genotyping

The samples were cleaned from foreign tissues. The DNA was extracted using the DNeasy Tissue Kit (Qiagen) and screened for the seven microsatellite loci that had been previously isolated from *S. lophyropoda *[52], under the amplifying conditions described in that paper. Alleles were sized on an ABI 3700 automated sequencer (Scientific and Technical Services of the University of Barcelona) using GENESCAN™ and GENOTYPER™ software for the genotyping.

### Identical multi-locus genotypes

Individuals sharing multi-locus genotypes (MLGs) could have resulted either from sampling individuals resulting from asexual reproduction (ramets), or from sampling two or more individuals resulting from sexual reproduction (genets). The probability of the latter depends on the allele frequencies in the population and the observed heterozygosities. To test this probability, we used the program MLGsim (10,000 simulations, P < 0.05; [53]). This program, using a simulation approach, calculates significance values for the likelihood that a MLG observed more than once in a population is the result of sexual reproduction.

### Population data analyses

The TRANSFORMER-3 program [54] was used to generate the input files for the software packages used. Within each population, the analyses were performed at the genet level (i.e., excluding those individuals that presented significantly low probability (P < 0.05) of having a sexual origin). Allele frequencies, the observed and mean number of alleles per locus [55], and the observed and expected heterozygosities were estimated using POPGENE [56]. Wright's [57] inbreeding coefficients, F_IS_, as in [58] and departures from the Hardy-Weinberg equilibrium (HWE), using Bonferroni corrections, were estimated using FSTAT version 2.9.3 [59]. Ewens-Watterson tests of neutrality (10,000 simulated samples) and linkage disequilibria among all pairs of loci were also tested with POPGENE [56] and GENEPOP [60], respectively.

Differentiation statistics based on microsatellite allele data are widely used for studying moderately-structured populations [61]. Wright's [57] F_ST _values (as in [58]) were estimated using ARLEQUIN [62] and Slatkin's [63] global R_ST _value (as in [64] Michalakis & Excoffier 1996) was estimated using GENEPOP [60]. Isolation by distance was tested using the correlation between pairwise F_ST_/(1-F_ST_) values and the logarithm of the geographical distances between the populations (as calculated from the minimum distance by sea), using a Mantel test. The analysis of molecular variance (AMOVA) is a testing procedure based on permutation analysis for studying molecular variation within species. It was performed using ARLEQUIN software [62]. The number of genetically homogeneous groups (K) was inferred using a Bayesian algorithm (STRUCTURE) [65]. K was inferred using the *ad hoc *statistic Δ*K *as in Evanno *et al*. [66]. The probability of recent effective population size reductions from allele data frequencies was tested using BOTTLENECK software [67]. This program is based on the assumption that populations, which have experienced a recent reduction of their effective population size, exhibit both a reduction of the allelic diversity and heterozygosity, although the allele numbers are reduced faster than the heterozygosity. The software computes for each population sample and for each locus, the distribution of the heterozygosity expected from the number of alleles, given the sample size under the assumption of mutation-drift equilibrium.

## Authors' contributions

AB conceived the study, performed the samplings, carried out the DNA genotyping, performed the statistical analyses, and drafted the manuscript. MJU also conceived the study and performed the samplings, supervised the genetic studies, and contributed to the writing of the manuscript. Both authors read and approved the final manuscript.
